# Consumption of Energy Drinks among Undergraduate Students in Taiwan: Related Factors and Associations with Substance Use

**DOI:** 10.3390/ijerph14090954

**Published:** 2017-08-24

**Authors:** Yen-Jung Chang, Ching-Yi Peng, Yu-Ching Lan

**Affiliations:** 1 Department of Health Promotion and Health Education, National Taiwan Normal University, No.162, Section 1, Heping E. Rd., Taipei City 106, Taiwan; 2 Department of Senior Citizen Service Management, Minghsin University of Science and Technology, No.1, Xinxing Rd., Xinfeng Township, Hsinchu County 304, Taiwan; chingyi@must.edu.tw; 3 Department of Health Risk Management, China Medical University, No.91, Hsueh-Shih Road, Taichung City 404, Taiwan; yclan.cmu@gmail.com

**Keywords:** energy drinks, substance use, undergraduate students

## Abstract

*Background*: This study aimed to investigate the consumption of energy drinks and associated factors among undergraduate students in Taiwan. *Methods*: Data came from a cross-sectional survey conducted in 2015. Eligible participants completed a self-administered questionnaire assessing use and perceptions of energy drinks, tobacco, alcohol, and betel nut. *Results*: Among 606 surveyed undergraduate students, 24.8% reported consuming energy drinks in the past 30 days. The major reasons for use included keeping alert at work (48.7%), being curious about the products (32.0%), enjoying the flavor (31.3%), or preparing for school exams (26.7%). Among energy drink users, half have never read the nutrition label, and 15.3% reported that they had ever mixed energy drinks with alcohol. Most participants showed negative attitudes toward using tobacco, alcohol, or betel nut, while 54.1% reported positive attitudes toward consuming energy drinks. Being male, living away from parents’ home, tobacco use, alcohol use, and positive perceptions of energy drink’s effects significantly predicted energy drink consumption. *Conclusions*: In addition to exploring motivations of energy drink consumption in undergraduate students in Taiwan, the study findings indicated that energy drink consumption might relate to the use of tobacco and alcohol, which should be taken into account in substance use prevention programs.

## 1. Introduction

Caffeinated energy drinks (EDs) may contain high amounts of caffeine, which may cause adverse health effects, such as the increased risk of cardiometabolic diseases and poor sleep quality [1,2,3]. Adverse cardiovascular events have been reported after ingestion of EDs or caffeine overdose [4,5]. Furthermore, the combined use of caffeine and alcohol, which is trendy among young adults in certain countries, may be linked to increased risk of excessive drinking or accident events [6,7,8]. Recent studies also suggest that the intake of EDs is associated with higher impulsivity and risk-taking behaviors, such as substance use [6,8,9]. 

Possible adverse health effects have been documented in the literature, however, the safety of using ED products could be overlooked by the lay population. This issue is particularly alarming in vulnerable groups, including children and adolescents. Adolescents and young adults younger than 25 years have been the main targets of fast-growing ED market in Western countries [2,10,11]. In Taiwan, imported and locally manufactured EDs are commonly accessible and available to all age groups, since the sale of EDs is not strictly regulated to date, while is been regulated or restricted in certain countries over concerns about their high caffeine content. Those regulations include labeling “high caffeine content”, suggesting a maximum daily consumption, indicating that EDs should not be mixed with alcohol, or even prohibiting their sale [1,12,13]. The consumption patterns of EDs and the hazardous use of alcohol mixed with EDs amongst undergraduate students in various countries have been well documented [3,6,11,14,15,16,17]. However, little is known about Taiwanese adolescents’ and young adults’ consumption of EDs, particularly undergraduate students who are the primary targets of ED advertising campaigns. In Taiwan, there are previous studies addressing the use of EDs among manual skilled workers [18,19], but there is limited data on the consumption of EDs among undergraduate students. 

This study aimed to investigate the consumption of EDs and associated factors among undergraduate students in Taiwan. The objectives of this study were: (1) to investigate the patterns and motivations of ED consumption among undergraduate students in Taiwan; (2) to explore the socio-demographic characteristics and lifestyle factors associated with ED consumption among undergraduate students in Taiwan, and (3) to examine the associations between ED consumption and substance use behaviors among undergraduate students in Taiwan. Chewing betel nut, an addictive substance that is carcinogenic to the oral cavity, pharynx, esophagus, liver and uterus [20], is also a risky behavior, in addition to smoking and drinking, widely discussed in Taiwan [21,22].

## 2. Materials and Methods

### 2.1. Study Participants and Data Collection

We conducted a cross-sectional study in Taiwan in 2015 and collected the data using an anonymous, self-administered pen-and-paper questionnaire. Eligible participants included undergraduate students at least 20 years of age or older, since parental consent is required for those under 20. Study participants were recruited from selected universities or vocational colleges in four cities in northern, central, and southern areas in Taiwan (i.e., two schools in Taipei, two schools in Hsinchu, two schools in Taichung, and one school in Tainan). Survey was conducted during the period between the beginning of semester and mid-term session. No incentive was provided to participants. The Institutional Review Board of the National Taiwan Normal University approved the study procedure and materials for this study (#201504HM006). Research staffs explained the study purpose and inclusion criteria to the potential participants verbally, allowed them to read information sheet, and obtained oral informed consent from study participants. 

### 2.2. Measurement

The questionnaire included four sections. The first section asked study participants about socio-demographics, self-reported height, weight, health status, sleep quality. The second section assessed the consumption of EDs, tea, coffee, tobacco, alcohol, and betel nut in the past 30 days. Participants were asked, “During the past 30 days, how often did you drink ED? Never, ≤1 time/week, 2–3 times/week, 4–5 times/week, or ≥6 times/week (every day or almost every day)”. Those who consumed at least one energy drink during the past 30 days were defined as ED users in this study [16,17], and they were asked to complete the third section of questionnaire concerning patterns and reasons of ED use. Those ED users were also asked whether they have ever read the nutrition label of ED products, whether they are aware of the effective contents of ED products, and whether they have ever used EDs combined with alcohol. The EDs in this study included imported (e.g., Red Bull) and locally manufactured (e.g., Raging Bull) products available for purchase in Taiwan at the time of survey, and these products are promoted as functional beverages with effects that help individuals stay energized or focused. The ED products included in this study were listed in the questionnaire to help participants understand the survey scope and answer questions. 

The fourth section was to assess the attitudes toward using ED, we asked participant’s perceptions of EDs’ potential helpfulness, including keeping alert, improving academic, work, and sport performance, improving physical health, and promoting social relationship (Cronbach’s alpha = 0.89). Likert scaling was employed and the respondent had four options, ranging from “strongly agree” to “strongly disagree”. We also asked participant’s respective perceptions of the acceptability of using EDs, tobacco, alcohol, and betel nut. 

### 2.3. Statistical Analysis

The data were analyzed using SAS version 9.3 (SAS Institute Inc., Cary, NC, USA). Descriptive analysis was performed to generate the distribution of the variables. One-way ANOVA and Chi-Square test were done to test the differences in covariates of interest between ED users versus non-users. Logistic regression was conducted to identify predictors of ED use. The significant *p*-value was set at 0.05 or less.

## 3. Results

### 3.1. Characteristics of Participants and ED Consumption

After excluding three participants with missing data or contradictory responses, the analyses included 606 undergraduate students from selected schools in four cities in Taiwan. Study participants included 48.0% male. The mean age was 23.1 (SE = 4.9), and more than half (53.8%) were full-time students. 

Among surveyed undergraduate students, 51.8% reported ever consuming EDs, and 24.8% reported consuming EDs in the past 30 days. Males were more likely to use EDs compared to females (37.6% versus 18.3%, *p* < 0.001). No difference in ED use was observed between university and vocational college students. On average, ED users had higher BMI compared to non-users (22.7 versus 21.5, *p* < 0.05). ED users were more likely to drink tea and coffee compared to non-users. In terms of preferred daily routine, ED users tended to consider themselves as “evening type” rather than “morning type” compared to non-users. However, no difference in self-reported sleep quality emerged between two groups (Table 1).

### 3.2. Reasons and Patterns of ED Consumption

Table 2 shows the major reasons for consuming EDs, including keeping alert at work (48.7%), being curious about products (32.0%), enjoying the flavor (31.3%), or preparing for school exam (26.7%). On average, 14.0% of ED users consumed 2–3 times per week and 3.3% consumed 4 times or more per week. The average age of the first time ED drinking was 15.7; and 63.5% of ED users had their first ED before 18 years old. Among ED users, half (50.7%) have never read the nutrition label on the product package, and 48.7% were unaware of the effective contents of EDs. Additionally, 15.3% of ED users (3.8% of the overall sample) reported ever mixing EDs with alcohol. 

### 3.3. Substance Use

Overall, 11.2% of the study participants reported smoking, 31.2% had used alcohol, and 1.2% had used betel nuts in the past 30 days. Furthermore, the intake of EDs in the past 30 days was significantly associated with the use of tobacco, alcohol, and betel nut. The ED users were more likely to use these substances compared to non-users. 

### 3.4. Attitudes toward Using EDs and Substances

Most surveyed participants showed negative perceptions of the acceptability of using tobacco (93.7%), alcohol (74.3%), or betel nut (92.9%) while fewer than half (45.9%) reported negative perceptions of consuming EDs (Table 3). For the perceived helpfulness of ED consumption, ED users tended to consider that the consumption would improve alertness, boost academic, work, and sport performance, and promote health and social relationship, compared with non-users. An overall score was computed to reflect the level of positive perceptions of EDs’ helpfulness. The average score of perceptions of EDs’ helpfulness was higher in the ED users than in the non-users (2.3 versus 2.0, *p* < 0.001).

### 3.5. Predictors of ED Consumption

Using multivariate logistic regression, we examined predictors of ED consumption in the past 30 days (Table 4). Variables that were significant at the bivariate level, in addition to age, were included in the logistic regression model. 

The results showed that being male (OR = 2.0, 95% CI = 1.2–3.3), living away from parents’ home (OR = 1.8, 95% CI = 1.3–2.8), using tobacco (OR = 2.0, 95% CI = 1.3–2.9), using alcohol (OR = 2.1, 95% CI = 1.1–4.1), and positive perceptions of ED’s effects (OR = 2.3, 95% CI = 1.5–3.7) significantly predicted the ED consumption in the past 30 days. The type of preferred daily routine (i.e., “evening type” or “morning type”) was not a significant predictor in the model.

## 4. Discussion

Among surveyed undergraduate students in Taiwan, 51.8% had ever consumed EDs in their lifetimes, and 24.8% reported consuming EDs in the past 30 days. Additionally, 15.3% of ED users reported that they have ever mixed EDs with alcohol. Major reasons for ED use can be work- or school-related (i.e., to keep alert at work and to prepare for school exam) and personal (i.e., to be curious about products and to enjoy the flavor). Although half of ED users were unaware of the effective contents or never read the nutrition label on product packages, surveyed students demonstrated relatively positive attitudes toward ED consumption. Other observed predictors of ED consumption included being male, living away from parents’ home, tobacco use, alcohol use, and positive perceptions of EDs’ effects.

Our findings indicated that about one-fourth Taiwanese undergraduate students had consumed EDs in the past 30 days, which is lower than the prevalence rates reported in other countries [11,14,15,16,17]. This difference in prevalence rates could be expected because EDs are a relatively new type of beverage in Taiwan, and consuming tea, also a caffeinated beverage, is popular in Taiwanese culture. In addition to consuming EDs, our data showed that 91.3% of surveyed participants had consumed tea in the past 30 days. Taiwanese undergraduate students may frequently consume tea and intake caffeine from other sources in addition to ED products. Future research may address whether the co-use of EDs and other caffeinated beverages increases the risk of caffeine overdose and related cardiovascular events. 

Alcohol mixed with energy drinks (AmED) is more hazardous compared to using either alcohol or energy drinks alone [23]. The previous literature reports that AmED relates to the use of tobacco, marijuana, and other stimulants [24] and leads to other undesirable consequences, such as increased alcohol consumption, risky sexual activities, and illegal driving [7,25,26,27,28]. Although the neurobiological mechanisms underlying the interaction between alcohol and EDs remains unclear, it was found in an animal study that chronic AmED consumption may cause an inflammatory response, oxidative stress, and cell death in the temporal cortex and hippocampus of rats [29]. In our study, we observed the use of AmED (15.3% of ED users) and propose that it is concerning, since currently there are no regulations or warning labels to limit the mixed use of EDs and alcohol in Taiwan.

To our knowledge, this is the first study in Taiwan investigating ED consumption among undergraduate students and emphasizing the importance of the association between ED use and substance use, which should be considered in future interventions targeting prevention or screening of substance use among undergraduate students. Furthermore, in the multivariate regression model, living away from parents’ home could link to the higher odds of ED use. In undergraduate students, to live away from parents’ home may imply less parental supervision, which might also be associated with the changes in lifestyle and risky behaviors such as substance use. Parents of adolescents were usually sources of information on the adverse health effects of EDs and disapproval of ED use [30]. Moreover, our data indicate that ED users tended to perceive that EDs help promote social relationship, suggesting that the social explanations of ED and substance use, such as the influences of peers, could be critical for undergraduate students. Future research may continue to address social influences of ED use and explore factors associated with the changes in ED consumption patterns or amount.

While the association between ED use and sleep quality was not identified in our study, we found that to keep alert at work was the most common reason of ED use, which might imply that undergraduate students drink EDs to stay focused or energized. Furthermore, in the study sample, half of ED users were unaware of the effective contents in EDs while demonstrating relatively positive attitudes toward ED consumption. 22% of ED users even showed positive perceptions of EDs’ health benefits. We also observed that approximately one-third of ED users reported that they consumed EDs due to curiosity, which was also suggested by a recent study in Canada [31]. Future research may examine the effects of ED advertising on the consumption motivations in youth and young adults. Our data show that the consumption of EDs may start at a young age, which might indicate the necessity of health education targeting young adolescents in addition to parents. 

We acknowledge several limitations of this study. First, the causality between predictors and ED consumption could not be established in this cross-sectional study. Second, the study population comprised a convenience sample of students from selected universities or vocational colleges in Taiwan; thus, it may not be nationally representative. Information on participant’s study faculty was unknown, but we recruited participants in the classes of general education courses in order to improve the study faculty variance. Moreover, the study sample is limited to those 20 years of age and older, which biased the sample to an older group of undergraduate students. Recall bias of the self-administrated questionnaire is also possible. We suggest that future studies recruit a representative sample and develop more comprehensive measurements.

## 5. Conclusions

The findings of this study indicated that being male, living away from parents’ home, tobacco use, alcohol use, and positive perceptions of EDs’ helpfulness significantly predicted ED consumption in the past 30 days. It is alarming that the consumption of caffeinated EDs in undergraduate students seems to be linked with risky substance use behavior, suggesting a need for future health education and research on substance use prevention in this context. Health interventions that consider the awareness of EDs’ contents and cautious use among undergraduate students are recommended.

## Figures and Tables

**Table 1:** Demographics, self-reported health, lifestyles, and substance use, by energy drink use in the past 30 days.

Characteristics	Use (n = 150)	No Use (n = 456)	Total (n = 606)
Male, % ***	50.7	27.6	33.0
Mean age (SD)	23.4 (5.5)	22.9 (4.7)	23.0 (4.9)
School type, %			
University	73.5	74.1	73.9
Vocational College	26.5	25.9	26.1
Employment status, %			
Not employed	54.0	53.7	53.8
Full-time employed	11.3	8.6	9.2
Part-time employed	34.7	37.7	37.0
Low household income, %	28.7	29.8	29.5
Live away from parents’ home, % **	56.7	42.7	46.1
Mean BMI (SD) *	22.7 (4.7)	21.5 (3.9)	21.8 (4.1)
Overweight or obese (BMI ≥ 25), %	8.8	8.1	8.3
Self-reported good physical health, %	29.3	26.8	27.4
Self-reported good mental health, %	50.7	45.8	47.0
Regular exercise, %	16.0	13.8	14.4
Self-reported good sleep quality—past 30 days, %	56.0	56.1	56.1
Type of preferred daily routine, % *			
Daytime	31.3	40.3	38.1
Nighttime	68.0	59.0	61.3
Frequency of tea consumption—past 30 days, % *			
No use	4.7	9.9	8.6
<4 times/week	44.7	47.7	46.9
≥4 times/week	50.7	42.4	44.5
Frequency of coffee consumption—past 30 days, % **			
No use	43.3	59.0	55.1
<4 times/week	45.3	31.8	35.2
≥4 times/week	11.3	9.2	9.7
Substance use-past 30 days, %			
Tobacco ***	22.7	7.5	11.2
Alcohol ***	48.0	25.7	31.2
Betel nut *	3.3	0.4	1.2

Note: *: *p* < 0.05; **: *p* < 0.01; ***: *p* < 0.001.

**Table 2:** Major reasons and patterns of energy drink use in the past 30 days.

Outcome	Total (n = 150)
Major reasons of use, %	
Keep alert for work	48.7
Be curious about the products	32.0
Enjoy the flavor	31.3
Prepare for exams	26.7
Frequency of use, %	
≤1 time/week	82.7
2–3 times/week	14.0
≥4 times/week	3.3
Mean age of the first ED use (SD)	15.7 (3.9)
Consumed the first ED before 18 years old, %	63.5
Had ever combined with alcohol use, %	15.3
Had ever read nutrition label on ED package, %	50.7
Had ever been aware of ED effective contents, %	48.7

Note: ED: energy drink.

**Table 3:** Attitudes toward using energy drinks and other substances, by energy drink use in the past 30 days.

Outcome	Use (n = 150)	No Use (n = 456)	Total (n = 606)
Positive perceptions of ED’s effects, %			
Keep alert **	72.2	61.8	66.1
Boost work performance **	43.3	27.9	31.7
Boost sport performance ***	36.7	20.6	24.6
Boost academic performance ***	28.7	13.6	17.4
Promote health **	22.0	11.6	14.2
Promote social relationships ***	20.0	8.3	11.2
Mean score of overall perceptions of ED’s effects ^a^ (SD), (range: 1–4) ***	2.3 (0.8)	2.0 (0.6)	2.1 (0.7)
Negative perceptions of use, %			
Energy drinks	45.3	46.1	45.9
Tobacco	91.3	94.5	93.7
Alcohol	76.0	73.7	74.3
Betel nut	90.0	93.9	92.9

Note: **: *p* < 0.01; ***: *p* < 0.001. ^a^ A lower score means a more negative perceptions of ED’s effects.

**Table 4:** Predictors of energy drink consumption in past 30 days.

Characteristics	Adjusted Odds Ratio	95% CI
Gender (male vs. female) ***	2.0	1.2, 3.3
Age	1.0	0.9, 1.1
Live away from parents’ home (yes vs. no) *	1.8	1.1, 2.8
BMI	1.0	0.9, 1.1
Score of perceptions of ED’s effects ^a^ (range 1–4) ***	2.0	1.3, 2.9
Type of daily routine (evening vs. morning)	1.5	0.9, 2.5
Drink tea—past 30 days (yes vs. no)	1.8	0.7, 5.1
Drink coffee—past 30 days (yes vs. no)	1.4	0.9, 2.2
Tobacco use—past 30 days (yes vs. no) *	2.1	1.1, 4.1
Alcohol use—past 30 days (yes vs. no) ***	2.3	1.5, 3.7
Betel nut use—past 30 days (yes vs. no)	2.6	0.3, 26.7

Note: * *p* < 0.05; *** *p* < 0.001. ^a^ A lower score means a more negative perceptions of ED’s effects. CI: Confidence Interval.
